# Research evidence and policy: qualitative study in selected provinces in South Africa and Cameroon

**DOI:** 10.1186/s13012-015-0315-0

**Published:** 2015-09-03

**Authors:** Celeste E. Naude, Babalwa Zani, Pierre Ongolo-Zogo, Charles S. Wiysonge, Lillian Dudley, Tamara Kredo, Paul Garner, Taryn Young

**Affiliations:** Centre for Evidence-based Health Care, Faculty of Medicine and Health Sciences, Stellenbosch University, PO Box 241, Cape Town, 8000 South Africa; South African Cochrane Centre, South African Medical Research Council, P.O. Box 19070, Tygerberg, Cape Town, 7505 South Africa; Centre for Development of Best Practices in Health, Central Hospital Yaoundé, University of Yaoundé, P.O. Box 87, Yaoundé, Cameroon; Division of Community Health, Faculty of Medicine and Health Sciences, Stellenbosch University, P.O. Box 241, Cape Town, 8000 South Africa; Liverpool School of Tropical Medicine, Department of Clinical Sciences, Liverpool, Merseyside L3 5QA UK

## Abstract

**Background:**

The translation of research into policy and practice is enhanced by policymakers who can recognise and articulate their information needs and researchers that understand the policymakers’ environment. As researchers, we sought to understand the policymaking process and how research evidence may contribute in South Africa and Cameroon.

**Methods:**

We conducted qualitative in-depth interviews in South Africa and focus group discussions in Cameroon with purposively sampled subnational (provincial and regional) government health programme managers. Audio recorded interviews were transcribed, thematically coded and analysed.

**Results:**

Participants in both countries described the complex, often lengthy nature of policymaking processes, which often include back-and-forth consultations with many diverse stakeholder groups. These processes may be influenced by political structures, relationships between national and subnational levels, funding and international stakeholder agendas. Research is not a main driver of policy, but rather current contextual realities, costs, logistics and people (clinicians, NGOs, funders) influence the policy, and research plays a part. Research evidence is frequently perceived as unavailable, inaccessible, ill-timed or not applicable. The reliability of research on the internet was questioned. Evidence-informed health decision-making (EIDM) is regarded as necessary in South Africa but is less well understood in Cameroon. Insufficient time and capacity were hindrances to EIDM in both countries. Good relationships between researchers and policymakers may facilitate EIDM. Researchers should have a good understanding of the policymaking environment if they want to influence it. Greater interaction between policymakers and researchers is perceived as beneficial when formulating research and policy questions as it raises researchers’ awareness of implementation challenges and enables the design of tailored and focused strategies to respond to policymakers’ needs.

**Conclusions:**

Policymaking is complicated, lengthy and mostly done at national level. Provinces/regions are tasked with implementation, with more room for adaptation in South Africa than in Cameroon. Research evidence plays a role in policy but does not drive it and is seen as mostly unavailable. Researchers need a thorough understanding of the policy process and environment, how the health system operates, as well as the priorities of policymakers. This can inform effective dialogue between researchers and policymakers, and contribute to enhancing use of research evidence in decision-making.

**Electronic supplementary material:**

The online version of this article (doi:10.1186/s13012-015-0315-0) contains supplementary material, which is available to authorized users.

## Introduction

Evidence-informed health decision-making (EIDM) is defined as an approach to decisions that ensures decision-making is informed by the best available research evidence. It is characterised by systematic and transparent approaches to access, appraise and use evidence as an input in the decision-making process [1]. Evidence-informed policies coupled with well-executed implementation and monitoring are likely to enable the achievement of health-related millennium development goals (MDGs) (e.g. reduce child mortality, improve maternal health, combat HIV/AIDS, malaria and other diseases), sustainable development goals (e.g. end hunger, achieve food security and improved nutrition and promote sustainable agriculture), as well as a reduction in burden of disease [2]. On the other hand, poorly informed decision-making may contribute to problems related to effectiveness, efficiency, costs and equity in health systems [1].

The decisions that policymakers make take the forms of laws, policies, regulations, guidelines and health promotion campaigns [3]. Policymakers, as a group, are generally not well defined; they differ in their levels of authority and roles and could be politically affiliated (elected) or neutral or performing purely bureaucratic functions (non-elected). Those who support policymakers in decision-making processes are equally diverse [4]. Often, policymakers are the technical people who lead the formulation of recommendations, policies and implementation decision, with more senior political figures turning to them for guidance on policy options to inform the final decisions. [5, 6].

Policymakers need robust evidence to clarify what services and programmes to offer, how to deliver them and how to implement change [2]. Evidence is required at various stages of the policymaking process: in defining the problem, assessing potential policy and programme options and identifying implementation considerations. At each of these policymaking steps, different types of evidence are required to inform decision-making [7]. Systematic reviews are well-recognised sources of best evidence [8]. They are more rigorous than traditional reviews and involve a serious attempt to reduce bias and statistical imprecision, thus minimising the risk of wrong conclusions [9, 8]. Policymakers need many types of systematic reviews to support policy formulation. Existing systematic reviews, overviews of systematic reviews and summaries of systematic reviews are available for policymakers to access and use.

Decision-makers often have limited capacity and resources for developing or supporting the development of evidence-informed policies and programmes [10]. Hindrances to EIDM include lack of time and skills required to acquire and appraise research evidence [11–13], unavailability of research at the time that it is required [13–15], irrelevance of research to the needs of decision-makers and presentation of research in formats that decision-makers cannot utilise [16–18]. The attitudes of decision-makers towards research and mistrust of researchers also hinder EIDM [11]. Reviews by Lavis et al. and Innvaer et al. found that personal interactions between researchers and healthcare policymakers and timing or timeliness of research increase the prospects for research use among policymakers, as does highlighting relevant information to policymakers and using structured summaries with clear recommendations [7, 11]. Importantly, the capacity of policymakers to recognise the need for research and communicate this need for research is one of the drivers that underpin knowledge translation activities, especially the user-pull strategies (where policymakers seek evidence) [19]. Additionally, a number of organisational-level factors can influence use of research evidence in decision-making. Factors that facilitate EIDM include technical infrastructure that ensures access to research evidence, establishing roles that actively stimulate research use, forming links to researchers and experts outside an organisation and capacity building of staff [20]. The numerous EIDM hindrances, facilitators, levels and contexts identified by researchers demonstrate the complex, interacting and multi-level nature of the implementation of research evidence by policymakers. This complexity has been conceptualised into a Consolidated Framework for Implementation Research (CFIR) by Damschroder et al., representing five major domains: intervention characteristics, outer setting, inner setting, characteristics of individuals involved and process of implementation [21]. The role of elements within these domains varies between contexts.

**Fig. 1:**
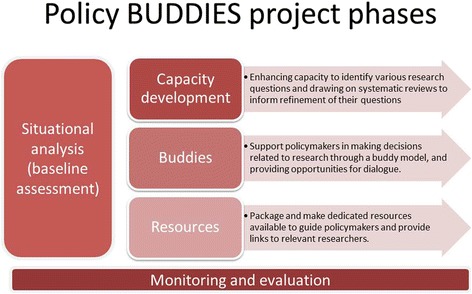
Policy BUDDIES—project phases

Use of research in health policy has been investigated in Tanzania and Malawi [22]; however, in South Africa (SA) and Cameroon, little is known about the implementation of EIDM at subnational level and the related capacity of policymakers to follow this approach when making decisions. Consequently, we embarked on a project called Policy BUDDIES—BUilding Demand for evidence in Decision-making through Interaction and Enhancing Skills Enhancing Skills (Fig. 1), with the ultimate aim of enhancing capacity in EIDM of researchers and policymakers. As phase 1, we conducted this situational analysis, to inform subsequent phases related to the development of appropriate initiatives to support policymakers and enhance links with researchers. The objectives of the situational analysis were as follows:

1. Describe the different contexts in which health policies are formulated and identify the facilitators and barriers to demanding research evidence.
2. Determine the roles, skills and resources of provincial health policymakers for supporting evidence-informed decision-making.
3. Determine priority areas for research and policymaking in provincial health departments.

## Methods

### Study design, population and setting

This qualitative study of health policymakers at subnational level used individual in-depth interviews in SA and focus groups in Cameroon. We defined policymakers as government officials working as health programme managers and programme coordinators at provincial level (or its equivalent) in SA and Cameroon, and purposively selected policymakers working in programmes related to MDGs 4, 5 and 6. In both countries, the public health system serves the vast majority of the population and there is a shift from curative, hospital-based health care to health services provided in an integrated manner within district health systems and using community-based approaches. SA (upper middle-income country) has a population of 2.5 times more people than Cameroon (lower middle-income country) (Table 1). Both countries have constitutional democracies. SA consists of three structures of government—national, provincial and local governments, and is divided into nine provinces, each with a provincial legislature. Provincial governments are bound by laws and policies passed at national level but can adapt or develop their own laws and policies within this framework to suit their specific needs. The priorities of national DoH for the period 2009–2014 were on meeting the MDGs, improving the quality of health services and the implementation of a National Health Insurance among others [23]. Cameroon is divided into 10 regions bound by laws and policies passed at national level, and has no authority to develop or adapt national laws or constitutions. Health care in Cameroon is guided by the Health Sector Strategy, which represents a holistic response by the Government of Cameroon to major health challenges and the need to protect and preserve public health.

**Table 1:** Country statistics (source http://www.who.int/countries/)

	Cameroon	South Africa
Total population (2013)	22,254,000	52,776,000
Gross national income per capita (PPP international $, 2013)	2660	12,240
Life expectancy at birth m/f (years, 2012)	55/57	56/62
Probability of dying between 15 and 60 years m/f (per 1000 population, 2012)	371/349	463/350
Total expenditure on health per capita (international $, 2012)	120	982
Total expenditure on health as % of GDP (2012)	5.1	8.8

### Sampling technique and enrolment

Policymakers were identified through engagement with specific disease clusters and programmes in government health offices in both countries. In SA, participants were purposively selected and identified through contact persons within the provincial DoH in two provinces. All available policymakers were invited to participate in interviews, and appointments with consenting participants were arranged telephonically and via email. When more than one participant from the same department consented, they were all enrolled. In Cameroon, participants were identified through engagement with the national Minister of Health, the Director of Human Resources in the national Ministry of Health and the Regional Delegates of Health, the head of health in the region. Four Regions were selected for this study by the Honourable Minister of Public Health in Cameroon. Following teleconferences, Regional Delegates agreed to convene meetings with Policy BUDDIES as the main or only item on the agenda. Officials were briefed on the project, and those who consented were invited to participate.

### Data collection

The interviews were conducted in rooms and offices within the participants’ working environments. An interview guide was used to guide discussions (Additional file 1). It drew on the Walt and Gilson policy analysis framework [24] and included questions on actors (the people who actually make programme policy in provinces), processes (how do they currently do this), context (hindrances and facilitators) and content. With a focus on programmes related to MDGs 4, 5 and 6, we selected specific policy processes to discuss, such as the prevention of mother to child transmission (PMTCT) of HIV, so that questions were not entirely theoretical and enabled policymakers to consider real examples. In SA, we collected data using in-depth interviews conducted by two trained investigators in English; one interviewer facilitated the interview while another took notes and contributed to the discussion when necessary. In Cameroon, we conducted four focus group discussions, one in each of the selected regions. Each focus group discussion was facilitated by the research team in both French and English. The interviews were captured using digital voice recorders, and additional field notes were taken by investigators to ensure full and accurate data capturing. Immediately after each interview and group discussion, interviewers compiled a summary of key points voiced during the interviews.

### Data management and analysis

Data captured with digital voice recorders during key informant interviews and focus group discussions were transcribed by trained transcribers for analysis. Audio files were transcribed verbatim, including any nonverbal or background sounds. Nonverbal sounds were typed in parentheses. The transcribers proofread all transcriptions against the audio file and revised the transcript file accordingly. All transcripts were audited for accuracy by the investigators who conducted the interviews or facilitated the focus group discussions. The names of participants did not appear on the transcriptions. Data analyses were conducted by the investigators. The transcripts were reviewed using the software ATLAS.ti version 7.1.3 [25] to identify emerging themes or patterns as they related to study objectives. After finalising themes, the transcripts were read and thematically coded and analysed. In addition, the summary notes taken by the interviewers were read to enhance the data from the recordings, especially if the interviewer documented any nonverbal communication that was not reviewable on the audio files. The quotes from the Cameroon data were translated from French by the investigators.

### Ethics and consent

Ethical approval was obtained from Stellenbosch University Health Research Ethics Committee (N13/02/021) and the national ethics committee for research on human subjects in the Ministry of Public Health in Cameroon. Permission to conduct the study was obtained from the DoH in SA and the national Ministry of Health in Cameroon. Informed consent was obtained from all participants.

## Results

In SA, we conducted nine interviews with 10 participants (one interview with two participants) employed in provincial DoH in two provinces. They were managers of a cluster of services or units, representing communicable diseases, non-communicable diseases, nutrition, strategy and support services. Their roles in policymaking processes included policy formulation, review, translation, adaptation and implementation and guideline development, adaptation and implementation, as well as monitoring of implementation. In Cameroon, we conducted four focus group discussions (7, 12, 18 and 9 participants per group in the 4 regions). They included representatives from reproductive health, health information systems, neglected tropical diseases, expanded programme on immunisation, planning, tuberculosis, monitoring and evaluation, malaria, nutrition, training and administration. The participants were mostly involved in the implementation of policies and strategies decided by the central level of the national Ministry of Health.

The section below details the findings, and Table 2 includes key quotes.

**Table 2:** Selected quotes

**Policymaking is complicated, iterative and takes time**
“The thing with policy development is, you’ve got to engage with a lot of stakeholders, clinicians, management, local level academics, people in programmes, you’ve got to draw from all of that”. South Africa
“But you need, to come together, all the parties, the community, health sector, and the decision makers, to understand and to have the same view on reasons why the coverage is low and to see together and adopt the same strategy and to look for the means, to adapt means to be used so as to address the issue at hand. The actors should be aware and try to have the understanding of the situation so as to adopt consensually the implementation strategies to deal with the problem at hand.” Cameroon
“Theoretically, national government should be making policy through consistent engagement with provinces to ensure that policies are aligned with realities on the ground, but in practice there is room for improvement”. South Africa
“…can take 2–3 months or a year and it depends on the person driving the policy in the province”. South Africa
**Research is not the main driver of policy**
“research there, plays a major role and the things, I mean the kind of work that Cochrane does is very important because what you do is you bring together… the world of literature and you can make those kind of policy recommendations…”. South Africa
“…at these meetings, evidence is not necessarily the major point or the major driver of a decision. It certainly plays a role, I suppose a massive role, but perhaps not the biggest”. South Africa
“…One gets the impression that research is not a priority in the Ministry of Health…One year ago we thought there was a positive move, with the creation of the documentation centre…but the centre seems to be a stillbirth…nothing has really changed.” Cameroon
“The expertise of the clinicians. They will have all the papers and the knowledge, it gets heavily tapped, especially in the HIV side, but there is a fair amount, in my impression, of opinion, you know, expert opinion, expert personal opinion that gets, makes its way up the chain”. South Africa
“…In health there is something which I will call NGO dictatorship. One gets the impression that they are the ones who call the shots, they are the ones who take decisions…they would say, I want that such a policy be put in place and I will finance it… and this will happen even without they do paying due attention to not consider implementation implications…”. Cameroon
“One gets the impression that nowadays, there are people at central level who have never been managers on the field, who do not know how a district health service works; yet they take decisions on how such operational levels should function…”. Cameroon
**Buy-in from both managers and “coalface” implementers plays a role in policy**
“the basic principle would be to try and take note of the ground voices and if they strongly oppose a particular piece of policy then we know, it’s going to be impossible to implement anyway”. South Africa
“…It is often difficult for the regional level to implement operational strategies decided at central level. For successful implementation, regions often adapt operational strategies developed at central level to conform to the local contextual issues including geography and availability of human resources”. Cameroon
“so there would be a look at the ideal and then it becomes policy and then no resources are allocated to that policy and you have to implement and you have to report on something that you don’t have the skills or the resources or the tools or the mechanisms to do and it just frustrates everybody and it actually undermines the ability of government to do better”. South Africa
**Research evidence is often unavailable, inaccessible, or not applicable or timeous**
“important thing, besides the length of it, the kind of layout, the kind of language, all of those things are very important. You’ve got to almost write it in plain English, not in the kind of jargon that full of statistical analysis and confidence limits and all of that.” South Africa
“Our limitation however is that the research that happens from the point of design, towards the end point, very often is divorced from the services”. South Africa
“Usually the way things happen in government is… things are supposed to happen like by yesterday” [but] “to generate the evidence usually takes a while”. South Africa
“Sometimes we go to surf in the internet, there is many website, as you continue, you should have contradiction of information that you need, so you have the lot and you don’t know how to do, you have many negative and many positive and finally you don’t know which one is reliable.” Cameroon
“…we are not able to download information from the Ministry of health online databases because there is limited internet access…”. Cameroon
“…There is no library, though there is internet…We sometimes use personal documents collated by colleagues…some colleagues have personal documents…because obtaining documents online is very expensive…”. Cameroon
**Good relationships and tailored methods of communicating evidence would potentially facilitate the adoption of evidence informed decision-making**
“There is no motivation, but I always say one needs a researcher to lean on. It is now more than 10 years that we have no formal links with the academic world…Even if one were to go to Yaoundé it is not evident that one would have access to big professors like you. I wonder how one can get access to researchers, through the Ministry of Health or through someone else who will hold one’s hand and introduce to the researchers?”. Cameroon
“Researchers should not be remote; should be “imbedded” in services to understand context and formulate questions”. South Africa

### Policymaking is complicated, iterative and takes time

SA participants described policymaking as a collaborative process involving engagement with many diverse stakeholder groups (actors). Depending on the policy, these include health professionals, managers in the department, academic institutions and community members. When appropriate, pharmacists, pharmacologists, clinical experts, interest groups (e.g. cancer or heart foundations), hospital chief executive officers and disease specialists would form part of committees. Similarly in Cameroon, the actors depend on the policy implementation issue being considered and involve the health sector as well as political leaders and the civil society.

The participants described the relationships between national and provincial/regional departments in the development and adaptation of policies. In both countries, policies are developed at national level but there are inter-country differences in whether they can be and are adapted at provincial/regional level. In SA, the national DoH is theoretically responsible for all health policy development and provinces are then tasked to adapt, translate and implement these policies based on local context, fiscal, practical and logistical realities, but that is not always the case. Policies developed at national level are not always adopted and implemented at provincial level in all provinces. The departments in the provinces have structures and committees that deal specifically with policy adaptation and development. At times, provinces deviate from national policies and develop their own policies, depending on availability of resources, expertise and needs identified at the provincial level. In Cameroon, however, policy development is more hierarchical and regions do not have constitutional authority to modify policies taken at central level of government but are tasked with contextualisation of implementation strategies.

We asked participants how policymaking processes are initiated and about the time it takes to formulate and finalise policies. In SA, the need for policy development at provincial level usually arises when policymakers realise that a policy is no longer working or needs to be reviewed or when a new policy is formulated and adopted at national level. In the process of developing, adapting or translating policies, a policy advisory group is convened. The role of this group is to consider the various policy options and, in doing so, draw on various sources of information (e.g. WHO documents, electronic literature searches, etc.), engage with universities and other experts and convene related meetings, policy discussions and workshops. In addition to drawing information from international literature, provincial departments can work with international bodies like WHO and UNICEF in the development of policies and typically have a pool of academics and clinicians they work with for each of the various fields. Consultations with national DoH, other provinces, clinicians and implementers (“ground voices”) also usually happen at this stage. The engagement between the advisory and the policymakers is usually iterative, and, depending on the implications of the policy under discussion, the entire process can take a long time to be completed.

Once final decisions have been made and the policy drafted, it is sent to managers and implementers to consider financial and other implementation implications. Policymaking and adaptation therefore involves making decisions about prioritisation, resource allocation and implementation on the ground, as well as monitoring to see if implementation is working. In Cameroon, implementation strategies are mostly developed locally during coordination or evaluation meetings at the regional delegation of health with district chief medical officers and these processes were also described as lengthy. When inter-sectorial collaboration between provincial health departments and/or other government departments (e.g. education or social development) is required, there is additional complexity. Furthermore, availability and structuring of funding may impact on the complexity of development and implementation of policies.

### Research is not the main driver of policy

In both countries, subnational policymakers described the sources they use to find evidence for informing policymaking. Participants from SA were more aware of systematic reviews being a source of synthesised best evidence. In Cameroon, the main sources of evidence relate to routine health information and decisions on implementation strategies were mostly based on best practices in highly performing districts and learning from failing districts. Surveys (demographic health surveys, household surveys, etc.) and routinely collected public health data (e.g. immunisation coverage data) are considered as the evidence on which the performance is measured, while coordination and evaluation meetings provide room for identifying bottlenecks and strategies tried by the highly performing districts. There is often no systematic scrutiny of the evidence for effective strategies. Consensus on what has worked in highly performing districts is generally suggested for trial in poorly performing districts without analysing other relevant factors such as cost effectiveness and cost benefits. Participants from Cameroon mostly referred to United Nations agencies (WHO, UNICEF, UNAIDS) and PubMed as primary sources of research evidence.

In both countries, research evidence is not considered the main driver of policy decisions. If managers do not buy into the policy change or new policy, they are able to block it, even if it is supported by best evidence. This is often related to opinions about the feasibility of implementing the policy on the ground, financial constraints related to implementation and expert opinion. Service specialists play key roles in policy advisory groups. In Cameroon, funding agencies and NGOs were identified as key role players in policymaking at both national and subnational levels.

Besides research evidence, in both countries, other factors that influence policy decisions include the current contextual realities, costs, logistics, practicality and the rights of people who will be affected by the policy. There are also political factors that play a role in decision-making. In SA, politicians like Premiers and Ministers may be receptive to using evidence that they understand, while other role players, for example the industry, sometimes lobby strongly to block evidence-based policy decisions that can negatively affect their profitability. In Cameroon, political interests at central level and UN agencies like WHO and UNICEF play a key role, but industry is mentioned among key players. In SA, for a final decision, policymakers often make a call, and they prefer to be aware of where “*the managers’ heads are*” with regards to that decision. On the other hand, in the case of Cameroon, the central level may take decisions without considering or consulting implementers.

### Buy-in from both managers and “coalface” implementers plays a role in policy

Participants in both countries described few factors that could hinder policy implementation including the critical role of buy-in by the various actors. In SA, excluding relevant people and not considering their buy-in when policies are being developed can create problems when policies have to be implemented. However, in Cameroon, buy-in from implementers was considered to be less important and regional managers have to find ways and means of implementing policies taken at central level, irrespective of whether they were consulted in the policy development or not.

Another hindrance mentioned by participants relates to adequate resources for new or adapted policies. At times, policymakers in SA do not accurately calculate resource implications for implementing the policy, and the lack of funds hinders implementation.

### Research evidence is often unavailable, inaccessible or not applicable or timeous

We asked the participants to describe the facilitating and hindering factors related to demanding and using evidence in policymaking. In general, the participants felt that policymaking is a difficult and vexed area, especially the application of evidence-informed policymaking. Despite this, some South African participants felt that a formal process of considering available research evidence should be part of all policy generation and translation activities. These policymakers highlighted that they do not have time to search the research databases for relevant evidence due to their workloads. Furthermore, research is often inaccessible, difficult and time-consuming to read, not applicable and ill-timed. They felt that presentation of evidence should be limited to one or two pages or 10 slides and should be written succinctly.

In addition, the evidence that is available may not be usable as it is often not well-aligned with the health services. Timeliness was also seen as a constraint to using evidence. Policymakers felt that sometimes, evidence is not available at the time they need it and that their timelines for decisions are mostly very short, often only a few days. Similarly, in Cameroon, hindrances to EIDM include the lack of human resources, capacities and skills to systematically document best practices from district level and to analyse routine health information, as well as limited internet access to search, identify and access relevant and reliable online resources. Regional policymakers in Cameroon expressed concern about the reliability of research evidence available on the internet. They outlined that the evidence from different research papers is often conflicting and one is at a loss of which one is reliable. This discourages the use of research evidence from the internet, coupled with the fact that the decision-makers have very limited time and some research articles are not free of charge.

In Cameroon, a handful of regional policymakers are familiar with EIDM as some participants have been trained in leadership and management. There is lack of incentives for operational research and EIDM at the regional level as the system is hierarchical, centralised and bureaucratic. These bureaucratic processes and scarcity of resources undermine supportive supervision of service delivery. Factors identified that are facilitators of EIDM include the willingness of policymakers to enhance skills in EIDM particularly in finding relevant and reliable online resources and critical appraisal of evidence for its adaptation, as well as the existing capacities and skills to analyse routine health information despite the poor quality of this information.

### Good relationships and tailored methods of communicating evidence would potentially facilitate the adoption of evidence-informed decision-making

To further describe factors that could facilitate the use of evidence in policymaking, the participants described the importance of relationships and suitable means of communicating research evidence to them. In SA, the efforts of researchers to push the evidence to policymakers are often not effective for informing policy. SA policymakers felt that these strategies should be preceded by relationship building and knowledge of the policymakers’ needs and priorities. As policymakers have limited time to read emails and often shelve emails for reading later but never return to them, they acknowledge that if they know the sender of the email and it comes at the right time, with a message that is aligned with their current priorities, they will immediately read it. They find summaries of research evidence useful and said that these should highlight key findings and shortcomings of the research and also provide recommendations. It should be tailored, focussed and relevant to priority areas for decision-making. The packaging of this information needs to be attention-grabbing and readily available. They were also open to having a repository with evidence summaries and policy briefs that are in their area of interest, although others indicated that having to search databases on their own may not be effective in assisting them to find and use research evidence.

In terms of methods to communicate evidence, some SA participants said they prefer to use emails instead of social media, as there are people without access to Facebook and Twitter. Many policymakers have smart phones and tablets but prefer to use social networks for personal use. In addition, the DoH does not allow access to social network sites within working hours or on the DoH network. Some participants, however, considered social networks as a possibility for knowledge translation activities. Face-to-face meetings between policymakers and researchers were considered by most participants to be more effective than emails or social media. Participants from Cameroon did not mention the use of social networks for communicating research evidence.

Good relationships between researchers and policymakers increase mutual respect and trust and promote conduct of relevant research, and for policymakers to take ownership of the research findings, researchers and policymakers should work together from the beginning of research studies and policymaking processes. This was more evident in SA, and in Cameroon, participants report very limited personal relationships with researchers. The participants lament the absence of links with academic institutions and do not know the best way to create such relationships, whether through their hierarchy in the central level of the Ministry of Health or through other means. Researchers should communicate research findings to policymakers, instead of policymakers only hearing about them from international conferences. Policymakers felt that researchers do not understand the health system and need to have better knowledge of policymakers’ needs.

Researchers should have a good understanding of the policymaking environment and process if they desire to influence it. Some policymakers were very keen to link to the researchers and have active engagement and dialogue with them. This dialogue and engagement will also enable researchers and policymakers to jointly think of and frame research- and policy-relevant questions. They can work together with mutual trust and in the process learn to understand each other’s environments. Additionally, policymakers felt that researchers should be aware of the implementation challenges that surround many policies. Researchers need better understanding of realities of decision-making complexities, randomness and constantly changing influences. Policymakers thus welcomed the proposed Policy BUDDIES strategy of linking a researcher with a policymaker as a two-way capacity and relationship-building strategy (“buddy” model). Policymakers were open to having a “buddy” that they can call on with their needs, while others felt that the person should be based in the DoH rather than academic institutions.

Regarding training, policymakers are generally open to research-related capacity development. They pointed out that researchers also need capacity development on the realities of the policymaking process. Policymakers requested training in finding and reading systematic reviews as well as in contextualising evidence. Decision-makers have diverse capacities for research ranging from persons with postgraduate research degrees to those with no research training or exposure at all. Training activities could be tailored to those with no research background, while also refreshing the knowledge of those with research training. Workshops should be arranged to take 1 day a week for a few weeks instead of requiring participants to be away from work for longer periods.

### Priority topics and research areas for policymaking

The policymakers highlighted several priority areas for research and policymaking in SA, which mainly centre on health system arrangements and implementation strategies for treatment and care of diseases as well as preventive strategies for infectious diseases, non-communicable diseases and injuries. Additionally, policymakers are seeking answers on health promotion, implementation of services, feasibility, acceptability adherence to medication and others. In Cameroon, priority areas mainly centre on community mobilization, health education through media, increasing the coverage of services, implementation strategies and retention of human resources. Their interest areas were around immunisation, maternal and child health, malaria and prevention of mother-to-child transmission of HIV.

## Discussion

We undertook a qualitative study with subnational level policymakers in SA and Cameroon to describe policy development and to determine the roles, skills and resources that policymakers have for supporting EIDM. Participants’ roles varied from policy adaptation, translation, implementation and monitoring in SA to mostly implementation in Cameroon. Policymaking was described as a collaborative process involving engagement with diverse groups of stakeholders. In both countries, policies are developed at national level but there are inter-country differences in whether they can be adapted at provincial or regional level. In the policymaking process in both SA and Cameroon, research is not considered as the major driver of policy decisions. Contextual realities, costs, logistics, practicalities and the rights of those who will be affected by the policies also influence policy decisions. The significance of context, highlighted as a major factor in our study, is aligned with the recognition that knowledge exchange and use processes at a collective-level (policy and organisational) are entrenched in organisational, policy and institutional contexts [26]. These contextual factors are captured in the CFIR as the outer setting (economic, political and social contexts where an organisation resides) and the inner setting (structural, political and cultural contexts through which implementation processes will proceed) [21]. In searching for evidence to inform policy decisions, participants in SA were more aware of EIDM. Research evidence is however perceived as not readily available, often inaccessible, difficult and time-consuming to read, not applicable or ill-timed and is often not aligned with needs. These findings are similar to those in other developing countries, where limited access to research and the format and interpretation of research findings were problematic [22]. The policymakers expressed that good relationships between policymakers and researchers and the researchers’ knowledge of policymakers’ needs and priorities could facilitate EIDM. Communication of evidence to policymakers should be summarised, simplified and tailored for their needs, as this was a consistent EIDM barrier described in our and other developing country studies [22]. Most of all, policymakers highlighted that researchers’ capacity must be enhanced to understand the policymaker environment.

Policymaking is influenced by interactions with various stakeholders [24]—as part of this collaborative process, researchers should aim to interact and collaborate as well. This is particularly important as policymakers usually have limited time to interact with researchers and interrogate research. Researchers should also understand that policymaking is not a linear process and should aim to be involved at different stages of the policymaking process [1], recognising the importance of all five domains in the CFIR [21] in the quest to better understand and develop effective knowledge exchange interventions at the collective-level. The duration of the policy development process varies for different policies. Often, at the point when policymakers and researchers interact, there is limited time for researchers to initiate and conclude research to timely inform policy development [11]. Therefore, policymakers and researchers should have ongoing engagement so that relevant research questions can be identified early in the policy development process. Furthermore, national governments should prioritise funding to support the conduct of locally relevant and applicable research and drive the research agenda [27, 28].

There is a need to raise awareness about EIDM in both countries. While it is important to build the capacity of policymakers to understand research, researchers need to build their own capacity to understand the policymaking environment if they desire to influence it. Efforts of researchers to push the evidence to policymakers should be preceded by relationship building and knowledge of the policymakers’ needs and priorities. The policymakers highlighted several priority areas for research and policymaking. In addition to their priority questions, policymakers are seeking answers on health promotion, implementation of services, feasibility and adherence to medication. Policymakers welcomed the proposed Policy BUDDIES strategy of linking a policymaker with a researcher as a two-way capacity and relationship-building strategy (“buddy” model). Policymakers were open to having a “buddy” that they can engage with in relation to their research evidence needs.

This study contributes novel knowledge on EIDM in SA and Cameroon and builds on the current knowledge on use of evidence in policymaking, with elements from the five major domains of the CFIR being identified as contributors to the implementation of research evidence by policymakers, particularly the outer and inner settings and the characteristics of individuals [21]. Importantly, this study highlights the need for researchers to be more aware of health system issues and to understand the policymaking process. Good relationships built through ongoing dialogue and engagement between policymakers and researchers are essential to aid EIDM. This will enable them to work jointly in thinking through relevant research and policy questions. In addition, researchers will be more aware of implementation challenges and more familiar with current policy issues, enabling them to respond to policymakers’ needs through tailored and focused strategies.

### Strengths and limitations

This study was conducted by trained investigators in English and French. Transcription and analysis were conducted separately for English and French interviews, and results were combined at the write-up stage. Only two provinces were included in SA, and in one province, only two participants were available to participate. Inter-provincial differences in health service delivery and resources are well accepted in SA, and consequently, the two provinces selected may not be representative of the situation in other SA provinces. Participants were willing to engage, and they could have already been in favour of EIDM. SA participants were aware that interviewers were affiliated with Cochrane and might have wanted to please interviewers. The data we obtained, however, did not seem to be biased towards EIDM.

## Conclusions

Researchers should strive for a thorough understanding of the policy processes, how the health system operates, as well as the priorities of policymakers. This can inform effective dialogue and engagement between researchers, those engaged in systematic reviews and decision-makers in the health services. Policymakers and researchers should think about policy and research questions together, including contextual issues regarding feasibility and implementation. Dialogues that promote links and relationships between researchers and policymakers have the potential to enhance the use of research evidence in decision-making.

## Additional file

Additional file 1:
**Interview guide.** (PDF 113 KB)
