# Supplementary material for: Research evidence and policy: qualitative study in selected provinces in South Africa and Cameroon
Source: Implement Sci. 2015 Sep 3;10:126. doi: 10.1186/s13012-015-0315-0 (PMC4557313; doi:10.1186/s13012-015-0315-0)
Supplement: Additional file 1: — Interview guide. (PDF 113 KB) [file 13012_2015_315_MOESM1_ESM.pdf]

ADDITIONAL FILE 1
Title: Research evidence and policy: Qualitative study in selected provinces in South Africa and Cameroon

INTERVIEW GUIDE

Introduce researchers

This study is being organised by Taryn Young and colleagues who work with Stellenbosch University.
The study is being funded through resources within the Policy BUDDIES project funded by the World Health Organisation.

Explanation of consent form

Before we begin, it is important for you to understand why the research is being done and what it will involve. We hope you have read the information sheet carefully. Is anything that is not clear?
You do not have to take part in this study. It is up to you to decide whether or not to take part. If you do decide to take part you are still free to withdraw at any time.

Explanation of process

The discussion will be tape-recorded and will then be typed out in an anonymous format, so that they can be analysed. This typed record of the conversation will be available for you to see if you wish and will be stored in a secure environment. You, or any comments you make, will not be personally identified in our report.

Record participant name, qualifications, how long they have worked in this department, their position and brief description on their job:
....
....
....
....
....
....
....

Background

1. What is a policy? Can you give us some examples of policies?
2. Can you give us some examples of how you have been involved with policy development? And with policy implementation?

We would like you to consider the current policy on the management of the prevention of mother to child transmission of HIV (PMTCT) *{other examples TB or maternal health policies can be selected based on the participants interests}*

Actors

3. Who was involved in the development of the PMTCT policy at provincial level?

4. What was your role in the PMTCT policy development?

Processes

5. How was the PMTCT policy developed? What process was followed?
6. Which information sources were used for policy making? *{Identify any existing framework for using evidence in policy making}.*
7. Did the department link with any organisations or individuals outside of the department? *{Explore the links between policymakers, research intermediaries and researchers. Existing relationships, frequency and forms of communication}*

Context

8. What was the relationship between the Provincial Department, the National Department of Health, the World Health Organization or other international bodies with regards to the PMTCT policy?

Content

9. What is evidence-informed policy?
10. How was evidence from research used to inform the PMTCT policies?
11. What is your view on the barriers and facilitators to the use of research evidence in policy making?

Upcoming policy questions

12. When was the most recent policy-planning exercise in your department?
13. When is the next policy-planning exercise in your department?
14. What are the priority policy issues for your department in the next 36 months?
15. What questions would you most likely seek answers for (governance, financial, delivery arrangements or implementation considerations, monitoring evaluation, quality improvement)?
16. There are a few tools to support the use of evidence in policy making and we would like to get your opinion on it.
    a. "Push strategies" (such as producing and disseminating summaries of systematic reviews),
    b. "Pull strategies" (increasing demand for evidence from policymakers)
    c. "Knowledge exchange" strategies involving on-going dialogue between researchers and policymakers

Explore use of face book and internet resources; cell phone
Explore need for capacity development
